# Predictive factors for false negatives following sentinel lymph node biopsy in early oral cavity cancer

**DOI:** 10.1038/s41598-022-10594-1

**Published:** 2022-04-28

**Authors:** Kouki Miura, Daisuke Kawakita, Isao Oze, Motoyuki Suzuki, Masashi Sugasawa, Kazuhira Endo, Tomohiro Sakashita, Shinichi Ohba, Mikio Suzuki, Akihiro Shiotani, Naoyuki Kohno, Takashi Maruo, Chiaki Suzuki, Takehiro Iki, Nao Hiwatashi, Fumihiko Matsumoto, Kenya Kobayashi, Minoru Toyoda, Kenji Hanyu, Yusuke Koide, Yoshiko Murakami, Yasuhisa Hasegawa

**Affiliations:** 1 International University of Health and Welfare, Mita Hospital, Tokyo, Japan; 2 Nagoya City University Graduate School of Medical Sciences, Nagoya, Japan; 3 Aichi Cancer Center Research Institute, Nagoya, Japan; 4 Osaka University Graduate School of Medicine, Osaka, Japan; 5 Saitama Medical University International Medical Center, Saitama, Japan; 6 Graduate School of Medical Science, Kanazawa University, Kanazawa, Japan; 7 Kushiro City General Hospital, Kushiro, Japan; 8 Juntendo University Graduate School of Medicine, Tokyo, Japan; 9 University of the Ryukyus Faculty of Medicine, Okinawa, Japan; 10 National Defense Medical College, Tokorozawa, Japan; 11 Kyorin University School of Medicine, Mitaka, Japan; 12 Nagoya University Graduate School of Medicine, Nagoya, Japan; 13 Graduate School of Medicine and Faculty of Medicine, Kyoto University, Kyoto, Japan; 14 Kurashiki Central Hospital, Okayama, Japan; 15 National Cancer Center Hospital, Tokyo, Japan; 16 Toyoda ENT Clinic, Maebashi, Japan; 17 Chukyo Hospital, Nagoya, Japan; 18 Nagoya Medical Center, Nagoya, Japan; 19 Asahi University Hospital, 3-23 Hashimotocho, Gifu, 500-8523 Japan

**Keywords:** Cancer, Medical research, Oncology, Risk factors

## Abstract

Prophylactic elective neck dissection (ND) with navigation surgery using radioisotope-based sentinel lymph node biopsy (SLNB) is non-inferior to elective ND in terms of survival but has an advantage in postoperative functional disability. We conducted a subgroup analysis to identify predictive factors for false-negative (FN)-SLNB in patients with early oral cavity cancer. This study is a supplementary analysis using the dataset of a previously reported randomized clinical trial on SLN navigation surgery for oral cancers. This study investigated the association of clinical and SLN-related factors with false-negative cases in the SLNB group. From 2011 to 2016, 275 patients were enrolled and randomly assigned to the ND and SLNB study groups, with 134 patients assigned to the SLNB group. In the SLNB group, seven cases with negative SLNs and neck recurrences were judged as FN-SLNBs according to the general definition. The number of detected SLNs with and without adjusting for the propensity score was significantly associated with FNs in the logistic analysis. FN-SLNB was associated with the number of identified SLNs, suggesting the need for careful postoperative monitoring for neck recurrence in patients with one or two identified SLNs after acquiring sufficient experience in the identification technique.

## Introduction

Treatment of early oral cavity cancer involves elective neck dissection (ND) in patients with a higher potential for cervical lymph node (LN) metastasis, while therapeutic ND is performed in patients with an originally low potential for cervical LN metastasis after the appearance of late metastases^1^, resulting in over and under operation, respectively. Therapeutic radical ND often results in functional problems. Although elective ND reportedly preserves function, standardized elective ND is less comprehensive and does not address individual potential metastases. Elective ND has a specific risk of skip metastasis, which potentially causes late metastasis. Crean et al.^2^ performed an extended supraomohyoid ND, and dissected level I‒IV lymph nodes in N0 oral cavity squamous cell carcinoma (OCSCC) cases, reporting a 10% potential of metastasis to level IV. Both elective ND and the wait-and-see policy of therapeutic ND have advantages and disadvantages regarding prognosis and function^3^.

Therefore, in our previously reported randomized controlled non-inferiority phase III trial of sentinel lymph node (SLN) navigation surgery in oral cancers^4^, we verified and reported that elective ND with navigation surgery based on radioisotope (RI)-based sentinel lymph node biopsy (SLNB) is non-inferior to uniform elective ND in terms of survival but has an advantage in postoperative functional disability, i.e., it is less invasive in patients with oral cavity cancer without clinical evidence of LN metastasis.

The results for the secondary endpoint of this trial showed approximately 15% false-negative (FN) biopsies in the SLNB group. To improve the accuracy of SLNB as an individualized therapy to ensure disease-free survival, a reduction in the FN results is necessary^5^. Therefore, further research must be directed towards achieving this. We conducted a subgroup analysis, using data from the same trial to identify the predictive factors for FN-SLNB in patients with early OCSCC.

## Results

### Clinical results of the SLNB group

From 2011 to 2016, 275 patients were enrolled and randomly assigned to the ND and SLNB study groups, with 134 patients assigned to the SLNB group, comprising the full analysis set. The 3-year overall survival (OS) rate was 87.9% (one-sided 95% confidence interval [CI], 82.4%) in the SLNB group^4^.

Of the 134 patients in the SLNB group, after excluding two patients who did not undergo SLNB and one patient with missing data on SLNB, 131 patients were eligible. Table 1 lists the clinical and SLNB-related factors. The clinical factors were sex, age, primary site, surgical technique, ND, operative time, blood loss, and pathological(p)TN; the SLNB-related factors were number of SLNs, positive SLNs, single-photon emission computed tomography (SPECT)/computed tomography (CT) (a fusion of functional images by SPECT and anatomical images by CT), and primary resection prior to SLNB. In the SLNB group, ND was performed for 52/131 (40%) patients with positive SLNs or pull-through resection, in which the oral cavity tissue was pulled to the neck for oncologic radicality, and the primary and neck tissues of level I-III nodes were resected en bloc. As a result, ND was performed in 44 patients with positive SLNs and 8 patients with negative SLNs by pull-through resection.

**Table 1:** Characteristics of the patients at baseline.

Characteristic	Sentinel node biopsy group (n = 131) number of cases (%)	
Age (median, range) -yr	63 (90–21)	
**Sex**		
Male	87	(66.4)
Female	44	(33.6)
**Site of primary tumor**		
Tongue	107	(81.7)
Other oral sites	24	(18.3)
**Surgical approach and extent of resection**		
Transoral	108	(82.4)
Pull-through*	23	(17.6)
**Neck dissection**		
None	79	(60.3)
Unilateral	47	(35.9)
Bilateral	5	(3.8)
**Operation**		
Time (median, range) -min,	167 (61–667)	
Blood loss (median, range) -gr,	30 (0–600)	
** Pathological TNM**		
pT1	53	(40.5)
pT2	69	(52.7)
pT3-4	9	(6.9)
pN0	86	(65.6)
pN1	24	(18.3)
pN2	21	(16.0)
**SLNB related factors**		
The number of nodes identified with the probe in each case (median, range)	3 (1–6)	
Positive SLN	44	(33.6)
* Scintigraphy*		
Planar imaging alone	50	(38.2)
SPECT/CT**	81	(61.8)
Primary resection prior to SLNB for shine through	20	(15.3)

*Pull-through: en bloc resection of the primary and neck tissues.

**SPECT/CT: single-photon emission computed tomography/computed tomography, the fusion of functional images by SPECT and anatomical images by CT.

SLN: sentinel lymph node; SLNB: sentinel lymph node biopsy; TNM: tumor, node, metastasis.

### SLNB and false-negative biopsies

Table 2 shows the number of SLN metastasis-positive, negative, and false-negative cases according to the number of SLNs identified with the gamma-probe and subsequently harvested. The median number of identified SLNs was 3 (range: 1–6), and the identification rate was 131/131 (100%). The rate of positive SLNs tended to increase with the number of identified SLNs, although with marginal significance (p = 0.057). In the SLNB group, seven cases with negative SLNs and neck recurrences were judged as FN-SLNBs according to the general definition (neck recurrence without SLN metastasis in oral cancer). One case with a negative SLN and a positive non-SLN, which was dissected during pull-through resection, may be considered equivalent to an FN, although it was not included in the study of predictors because its background differed from that of the others in terms of the absence of neck recurrence. Cases were divided into two groups according to the number of SLNs, with cases with one to two SLNs defined as the “few” group and those with three or more SLNs as the “medium–high” group. The group with few identified SLNs included 34 cases with negative SLNs and six FN cases, whereas the medium–high group included 53 cases with negative SLNs and one FN case. The FN rates in both groups were 6/(34 + 6) (15%) and 1/(53 + 1) (1.9%), respectively. The medium–high group had a significantly lower proportion of FNs than the few group using Fisher's exact test (p = 0.013).

**Table 2:** The number of detected sentinel lymph nodes (SLNs) per case with the corresponding number of negative SLNs cases, number of neck recurrences, and false-negative cases.

Number of SLNs detected	Positive cases	Negative cases	Proportion of positive cases (%) *	*p* value	False-negative cases (%) **	*p* value***
1	2	10	16.7	0.057	2 (20.0)	0.029
2	9	24	27.3		4 (16.7)	
3	10	24	29.4		0 (0)	
4	16	17	48.5		1 (5.9)	
5	5	10	33.3		0 (0)	
6	2	2	50.0		0 (0)	
1–2	11	34	24.4	0.109	6 (17.6)	0.013
3–6	33	53	38.4		1 (1.9)	

*Positive case/(positive cases + negative cases).

**False-negative cases/negative cases.

***False-negative cases and negative cases were used as categories of a 2 × 2 contingency table.

The trend of proportion was tested using the Cochran-Armitage test. Dichotomous SLN number and proportions of negative and false-negative values were tested using the chi-square test and Fisher’s exact test.

The clinical factors that are associated with a high number of detected SLNs were investigated (Table 3). The primary site, SPECT/CT, shine-through, and gamma probe were significantly associated with the number of SLNs detected. Among these, the primary site was most associated with the number of SLNs identified, based on odds ratios. A propensity score was calculated using the logistic regression model. The SLN number was significantly associated with the FN risk after adjusting for the propensity score (odds ratio: 0.08, 95% CI: 0.01–0.76, Table 4).

**Table 3:** Factors associated with the detection of a high number of SLNs.

Variables	Categories	OR	95% CI	*p*
Age	Years (continuous)	0.99	(0.97–1.02)	0.684
Sex	Male	Reference		
	Female	0.86	(0.36–2.06)	0.736
Primary site	Tongue	Reference		
	Others	0.20	(0.07–0.61)	0.005
Primary resection	Peroral	Reference		
	Pull-through	2.72	(0.80–9.22)	0.109
pT	pTis-T1	Reference		
	pT2	1.07	(0.44–2.61)	0.885
	pT3-4	0.47	(0.09–2.57)	0.385
SPECT/CT	No SPECT/CT (planar imaging alone)	Reference		
	SPECT/CT	2.60	(1.03–6.53)	0.042
Shine-through	No prior resection	Reference		
	Primary resection prior to SLNB	4.27	(1.22–14.90)	0.023
Gamma-probe	Probe A	Reference		
	Probe B	0.25	(0.10–0.61)	0.003

OR: odds ratio; CI: confidence interval; SLN: sentinel lymph node; SPECT/CT: single-photon emission computed tomography/computed tomography.

**Table 4:** Impact of detected SLN on false-negative results.

Variable	Category	OR	95% CI	*p*
Number of SLNs	1, 2	Reference		
	3 +	0.08	(0.01–0.76)	0.028

The number of SLNs was included in a logistic regression model as a covariate.

Propensity score analysis and inverse probability weighting were used to adjust the influence of clinical factors. A propensity of detection of three or more SLNs was assessed by logistic regression using age, sex, primary site, primary resection, pT, SPECT/CT, shine-through, and gamma-probe as covariates.

OR: Odds ratio; CI: Confidence interval; SLN: sentinel lymph nodes.

Multivariable analysis for the association between the FN risk and number of SLNs was conducted using clinical factors as covariates (Table 5). The number of SLNs was associated with the FN risk, which was consistent with the propensity score analysis result. Other variables were not associated with the FN risk. The variables in the model did not show correlations. The strongest correlation coefficient in the correlation matrix was -0.35.

**Table 5:** Association between clinical factors and false-negative results.

Variables	Categories	OR	95% CI	*p*
Number of SLNs	1, 2	Reference		
	3 +	0.03	(0.00–0.75)	0.033
Age	Years (continuous)	0.98	(0.92–1.04)	0.516
Sex	Male	Reference		
	Female	2.07	(0.29–14.99)	0.472
Primary site	Tongue	Reference		
	Others	2.71	(0.24–30.95)	0.423
pT	pTis-T1	Reference		
	pT2	0.34	(0.04–3.06)	0.335
	pT3-4	4.19	(0.22–81.20)	0.344
SPECT/CT	No SPECT/CT (planar imaging alone)	Reference		
	SPECT/CT	0.36	(0.04–2.96)	0.341
Shine-through	No prior resection	Reference		
	Primary resection prior to SLNB	16.97	(0.59–489.88)	0.099
Gamma-probe	Probe A	Reference		
	Probe B	5.72	(0.57–57.39)	0.138

OR: Odds ratio.

CI: Confidence interval.

SLN: sentinel lymph node.

SPECT/CT: single-photon emission computed tomography/computed tomography.

## Discussion

We previously reported that SLNB navigation surgery was non-inferior to elective ND in terms of prognosis and was functionally superior. However, the FN-SLNB rate was 15% in this phase III study, which must be reduced further. The few SLNs group had a low rate of SLN metastasis positivity and a high rate of FNs, indicating the importance of the SLN number.

The purpose of this study was to evaluate in more detail the results of our early analysis that identification of a smaller number of SLNs would result in more FNs. The number of SLNs identified was related to other clinical factors. These clinical factors should also be associated with FNs. Therefore, the probability of identifying more SLNs was adjusted as the propensity score, and the relationship between the number of SLNs identified and recurrence was shown. The relationship between FNs and clinical factors in the usual multivariate analysis was also revealed.

From this result, we may state that the prognosis is significantly better when the number of SLNs identified is large. Multivariable analysis without propensity score matching showed the same result.

In addition, as several factors reduce the number of SLNs identified in the initial procedure, it may be necessary to actively search for SLNs in cases in which the number of SLNs identified is expected to decrease. Therefore, we considered the factors that influence the migration of cancer cells to SLNs and their identification.

Factors that determine the spread of cancer to SLNs and the detection of SLNs are as follows: (i) the lymphatic network of the tumor and surrounding mucosa, and (ii) diagnosis-related parameters such as surgical techniques, the influence of the shine-through phenomenon, and optimum tracers.

First, the association of the spread of cancer to SLNs with the lymphoid network of the oral cavity and lymphangiogenesis in the peritumoral mucosa has been discussed in the research stage, especially the difference in subsites.

Inoue et al. investigated the development of lymphatic vessels in the oral mucosa and found that the new lymphatic vessels in OCSCC have endothelial cell characteristics inferred to be associated with early lymphatic development and initial dissemination of cancer cells^6^. Oral cavity cancer promotes lymphatic vessel growth, which may affect tumor spread to the SLNs and identification by tracers, but the difference between subsites is controversial. Consistent with these results, we also found that lymphangiogenesis to the SLNs may occur before cervical metastasis in OCSCC^7^.

The differences in the lymphoid networks between the tongue, gingiva, palate, and other subsites, as well as the biology of the lymphovascular growth factor of the tumor, may affect the identification of SLNs of the tongue compared to the rest of the oral cavity.

The next factor is diagnosis. Diagnosis-related parameters have been discussed and proposed in the guidelines of the 8th International Symposium on SLNB^8,9^.

First, to perform SLNB as a surgical technique including gamma probes, an important consideration is the skill developed at the individual and institutional levels. Two gamma detection systems were used for the gamma probe. Both probe types can be used intraoperatively, and their specifications, such as energy range and maximum count range, are similar, indicating that there is no apparent difference in their capabilities as devices. The choice of these devices was not personal but institution-dependent, and the experience of the institution may have been reflected in the results. Although there is no minimum number of cases required to be qualified to perform SLNB, we developed proficiency in this technique via the following steps before conducting the phase III study: (1) a surveillance study^10^, (2) a phase II study^11^, and (3) a phase III study^4^.

Utilizing the experience obtained from the previous retrospective and phase II studies, 14 of 16 institutions adopted and perfected the procedures for biopsy, dissection, and pathological diagnosis to be used in the phase III study.

Additionally, a problem of SLNB based on the RI method is the shine-through phenomenon, in which the high accumulation in the RI region masks the accumulation in the neighboring SLN. This may have increased the proportion of FN-SLNBs. As mentioned earlier, in the head and neck region, the effect of this phenomenon has been observed in cancer of the floor of the mouth. The same phenomenon also occurs in the sublingual mucosa and lower gingiva. In this study, we found that sites other than the tongue may be associated with the reduced number of identified SLNs in the SLNB group. Some reports suggest that SLNB based on the RI method is not indicated for the floor of the mouth^12^. Stoeckli et al. reported a technique to routinely investigate level I nodes with a gamma probe after submental and preglandular fat pad mobilization or dissection through a submandibular incision for floor of the mouth cancers^13^.

To counter the shine-through phenomenon, prior resection of the primary tumor, combined use of SPECT/CT, and use of other tracers, including indocyanine green (ICG) fluorescence, have been reported.

Prior resection of the tumor to reduce the shine-through may be useful to reduce the radiation signal; however, there is currently no evidence showing that this improves the accuracy of SLN localization^8^. Although the consequences of prior resection have not been fully investigated, it was a significant factor in our analysis. However, in our study, cervical recurrence was more common in the prior resection group than that in the non-prior resection group, although this has not been reported previously. This suggests that further investigation is needed for future indications.

SPECT/CT labeling based on anatomical landmarks is useful to detect SLNs. Recently, the advent of new technological advances (portable gamma cameras, free-hand SPECT devices, dedicated probes, and navigation tools) together with preoperative SPECT/CT has led to the refinement of the original procedures based on conventional gamma camera imaging and handheld gamma probe detection^9^.

In addition, ^99m^Tc-tilmanocept (Lymphoseek®), which is used in Europe and the United States, rapidly migrates from the site of injection to lymphatic vessels, and after migration to lymphoid tissues it binds to mannose-binding receptors (CD206) on the surface of macrophages and dendritic cells, and accumulates in lymphoid tissues^14^. Therefore, although it is an RI tracer, it is considered to have a small shine-through phenomenon.

For SLN detection, dyes are usually used as a supportive method for RI. ICG fluorescence imaging is a non-RI method with no radiation exposure and easy endoscopic injection into the hypopharynx, which may be useful to expand the range of SLNB indications^15^. The hybrid tracer indocyanine green-^99m^Tc-nanocolloid contains a radioactive and a fluorescence signature in a single tracer, making it possible to combine preoperative nuclear medicine imaging with intraoperative radio- and fluorescence guidance.

In addition, tracers may be either RIs or optical imaging systems; RIs are mainly used for head and neck cancer. Regarding colloids, the approved pharmaceuticals differ depending on the country. In Japan, tin colloid and phytate are approved and used, and phytate was used in this study.

To identify SLNs, an ideal tracer must be able to move from the injection site through the lymphatic channel and get trapped at the SLNs. The dispersion of small particles (< 100 nm) is necessary for translocation, and large particles (500–2000 nm) remain trapped at the injection site^16,17^. Phytate is not a colloid but combines with extracellular calcium to form colloids, and the particle size of phytate colloids, which ranges from 200 to 1200 nm, changes according to concentration^18^. Phytate is an ideal tracer to identify SLNs because, when injected, it is sufficiently small to translocate, and after reaching the SLNs, it is sufficiently large to get trapped at the nodes.

In the SENT study, a ^99m^Tc-nanocolloid (Nanocoll/Nanocis®) was used as a tracer at a median dose of 57 MBq, and the number of identified SLNs were 3.2 per patient (range: 1–10)^19^.

Nanocolloidal albumin (Nanocoll® and NanoTOP®) has a mean particle size of 5–80 nm, with a maximum of 100 nm, and the Rhenium sulfide nanocolloid (Nanocis®) has a mean particle size of 50–200 nm, with a maximum of 500 nm^9^.

Meanwhile, the number of SLNs identified at 74 MBq of phytate (mean particle size: 200–400 nm, maximum: 1200 nm)^9^ was 3.1 per patient (range: 1–6)^4^. Despite the differences in colloid size, there seems to be no significant difference in the number of SLNs identified with nanocolloids and phytate.

The FN rate for oral cancer reportedly ranges from 2.56% to 36%^14,20^. In a systematic review and meta-analysis of 98 reports in which the diagnostic accuracy of SLNB for oral cancer was evaluated, Kim et al.^21^ reported a sensitivity of 0.827 and a specificity of 0.981. They concluded that the high specificity supports the role of SLNB as a diagnostic tool for patients with early oral cancer. However, the FN rate, or sensitivity, should be further improved.

The numbers of SLNs and FNs in SLNBs of cancers besides oral cancer have also been discussed. Scoggins et al.^22^ reported an analysis of FNs in the melanoma trial, MSLT-1^23^. They calculated an FN rate of 10.8%, and discovered that FN results are associated with a higher patient age, lower mean tumor thickness, less frequent lymphovascular invasion, and greater risk of local/in-transit recurrence. Moncrieff et al.^24^, in a study of SLNB for melanoma, showed that the accuracy of determining SLN location in primary cutaneous melanoma could be greatly improved with the introduction of SPECT/CT.

In breast cancer, Martin et al.^25^ conducted a prospective, multicenter study of patients with early-stage cancer and performed a multivariate analysis of FNs. They reported FN rates of 13.7% for a single identified SLN and 5.4% for more than one. They concluded that surgeon experience, tumor size and location, and the number of SLNs removed are independent preoperative and intraoperative predictors of the risk of a FN result. Similarly, Wong et al.^26^ reported that the ability to identify multiple SLNs improves the diagnostic accuracy of SLN biopsy in a study of FNs in early breast cancer. The above results were similar to ours. Liu et al.^27^ performed a meta-analysis of tracers in breast cancer and showed that methylene blue (MB) + ^99m^Tc or MB + ICG mapping methods could yield a higher identification rate and lower FN rate than MB alone.

These results suggest the use of multiple tracers for mapping SLNs, as well as the use of devices such as SPECT/CT that are excellent for positional diagnosis.

Tracers, including the hybrid types, have already been discussed. We have also conducted a study^28^ using ICG fluorescence, but further studies are needed.

This study had some limitations. The first limitation was the small cohort size of FN SLNBs. Second, there are variations in equipment and experience of surgeons among institutions. We conducted a phase II trial before the phase III trial to standardize the procedures and improve the learning curve to minimize the variation in personnel and procedures.

The differences in equipment, especially the use of SPECT/CT, need to be understood by each institution performing SLNB and promoted as a standard diagnostic technique.

In conclusion, we found that FN-SLNBs were associated with the number of identified SLNs. Diagnostic imaging techniques such as SPECT/CT should be used to improve the diagnostic accuracy of the shine-through phenomenon. Careful postoperative monitoring for neck recurrence is needed in patients with one to two identified SLNs after the acquisition of sufficient experience in the identification technique, and elective dissection may be considered in patients with just a single identified SNL.

## Methods

### Compliance with ethical standards

The current study was a supplementary analysis of a previously reported clinical trial^4^. The clinical trial was a phase III, multicenter, randomized controlled trial conducted to evaluate the non-inferiority of SLNB navigation ND (SLNB group) versus elective ND (ND group) in T1 (depth of invasion ≥ 4 mm) -T2N0 OCSCC. It was registered in the UMIN Clinical Trials Registry (UMIN000006510) in November 2011. The clinical trial was approved by the ethics committees in each institution and performed under the Safety and Efficacy Evaluation Committee’s oversight. Written informed consent was obtained from all participants. This research was performed following the relevant guidelines and regulations, including the Declaration of Helsinki.

### Patient groups for analysis

In this study, 134 patients from the SLNB group of the phase III trial were included. The basic procedure of SLNB has been reported in detail in the phase II study^11^. The day before surgery, ^99m^Tc-phytate was prepared as a radiopharmaceutical (tracer), and 74 MBq (2 mCi) of 1 mLwas dispensed with a 27G needle into four locations (0.25 mL per location) in the peritumoral mucosa. Planar images were taken in five directions with a scintillation camera at 1 to 2 h after administration as standard imaging. In addition, SPECT was performed when possible, and fusion images of SPECT and CT were obtained.

On the day of surgery, before ND, the SLNs were retrieved using two types of gamma probes depending on the study center. Both of these gamma detection systems were probe-type devices, with energy ranges of 12–600 keV and 0–650 keV, respectively, and maximum count ranges of 90,000 and 99,999 counts per second, respectively. During surgery, 2-mm-thick blocks of rapid-frozen specimen were paraffin-embedded for a more detailed postoperative evaluation. ND was then performed based on the evaluation of the frozen section pathology.

The primary site was resected before SLNB at the judgment of the surgeon when an intraoral approach was possible, to avoid the shine-through phenomenon. Shielding was used at some study centers and was performed at the surgeon’s discretion.

For positive SLNB between levels 1 and 3, dissection of the ipsilateral level 1–4 region was performed, and for positive level 4 region, dissection of the ipsilateral level 1–5 region was performed. If the frozen-section pathology evaluated intraoperatively was found to be negative, an excisional biopsy was performed without dissection. In cases where a free or pedicled flap was required for reconstruction, supraomohyoid ND of the affected side or healthy side was used. If postoperative histopathological evaluation by hematoxylin and eosin and cytokeratin immunostaining revealed metastases, in principle, a two-stage ND was performed within 6 weeks, and isolated tumor cells^29^ were treated as positive metastases.

### Predictors for false-negative SLNB

The study aimed to elucidate clinical factors associated with false negatives.

FN cases were defined as neck recurrence after the histological results were negative for SLNs^8,30^. The FN rate was defined as FN/(true positive + FN).

### Statistical analysis

A contingency table was created using data from Fisher's exact test and the chi-square test according to the sample size and distribution. The trend in proportions was examined with the Cochran-Armitage test. The FN risk was evaluated by odds ratio, and the 95% CI was calculated by logistic regression. To avoid confounding, a propensity score was used. A propensity of detection of three or more SLNs was assessed by logistic regression using age, sex, primary site, primary resection, pT, SPECT/CT, shine-through, and gamma-probe as covariates. The association between the numbers of FNs and SLNs was assessed by logistic regression using propensity scores with inverse probability weighting. To perform logistic regression analysis, we categorized the variables. Age was used as a continuous variable; sex was subcategorized into male and female, primary site into tongue and others, primary resection into peroral and pull-through, pT into pTis-T1, pT2, and pT3-4, SPECT/CT into none and with SPECT/CT, shine-through into none and prior primary resection, gamma-probe into probe A and probe B, and the number of SLNs into one or two and three or more. All statistical analyses were performed using STATA ver. 15.1 (STATA Corporation, College Station, TX, USA). A *p *value less than 0.05 was considered statistically significant.

## Data Availability

Since patient data cannot be made available, no access details can be provided. Any other requests for information should be made to the corresponding author.
